# Supplementary material for: Characteristics and weathering mechanisms of the traditional Chinese blue brick from the ancient city of Ping Yao
Source: R Soc Open Sci. 2020 Aug 19;7(8):200058. doi: 10.1098/rsos.200058 (PMC7481719; doi:10.1098/rsos.200058)
Supplement: Examples of intact blue brick samples; All pore size distribution curves; Failure photos and UCS values; Plots used for the experimental determination of the maximum firing temperatures; Temperature and rainfall/snowfall during moisture testing period [file rsos200058supp1.doc]

**Supplementary material**

**S1 Examples of intact blue brick samples.**


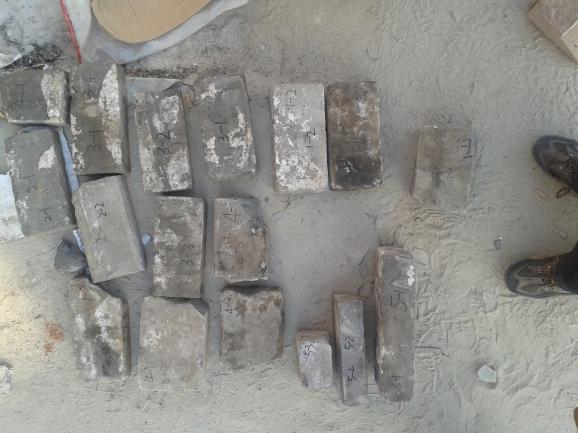

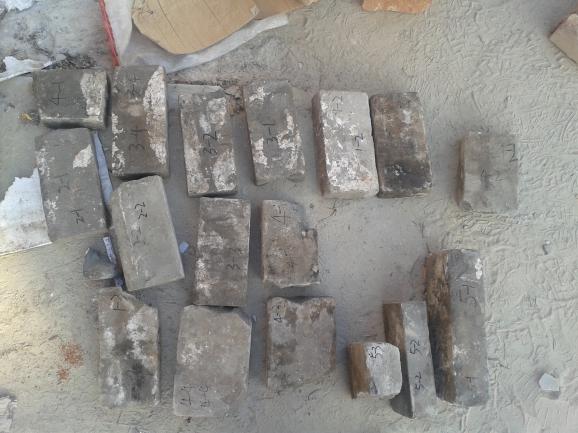


**Fig. S1 Photos of intact blue brick samples.**

**S2 All pore size distribution curves.**

Brick:

Hg-1:


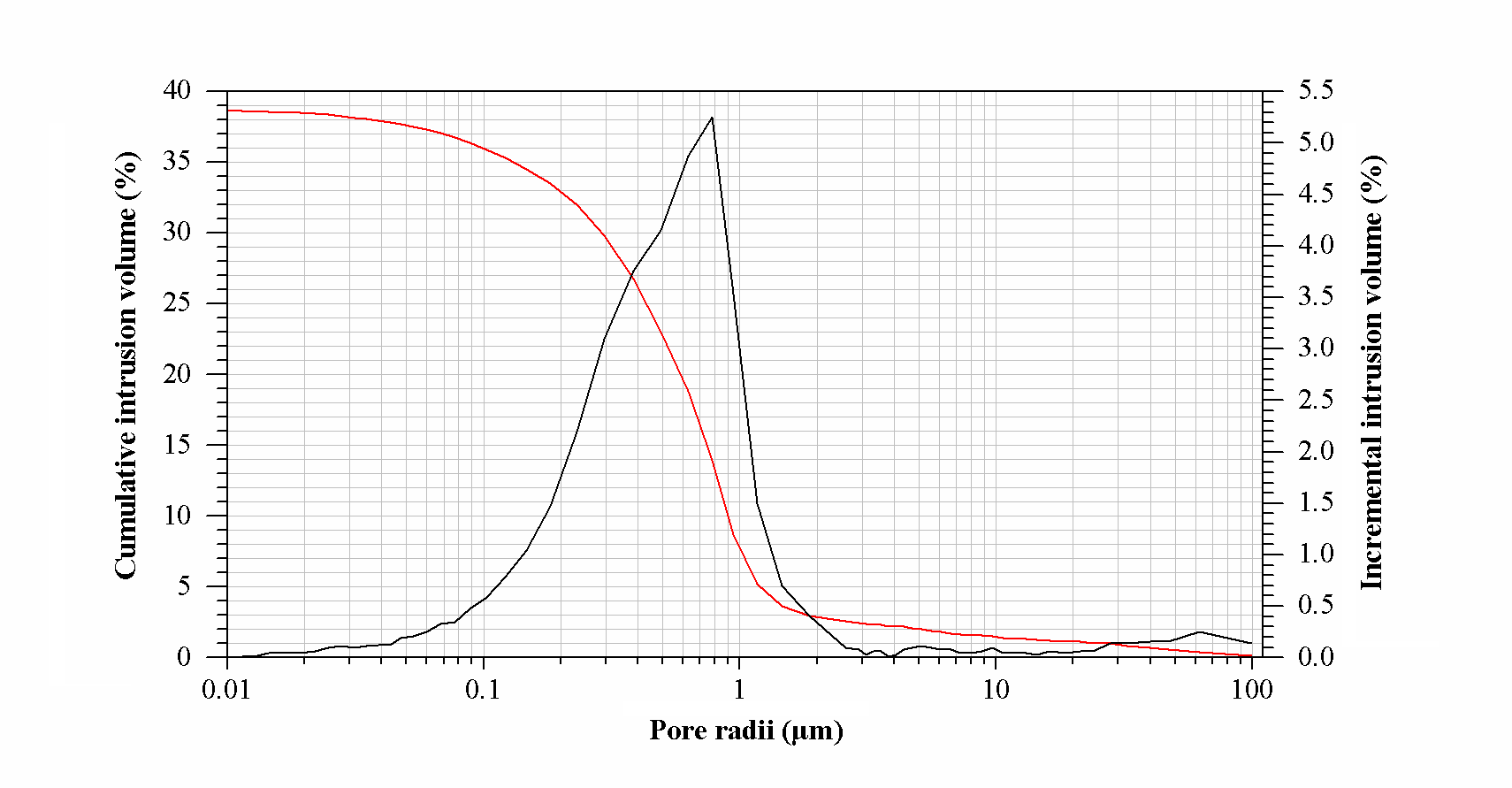


Hg-2:


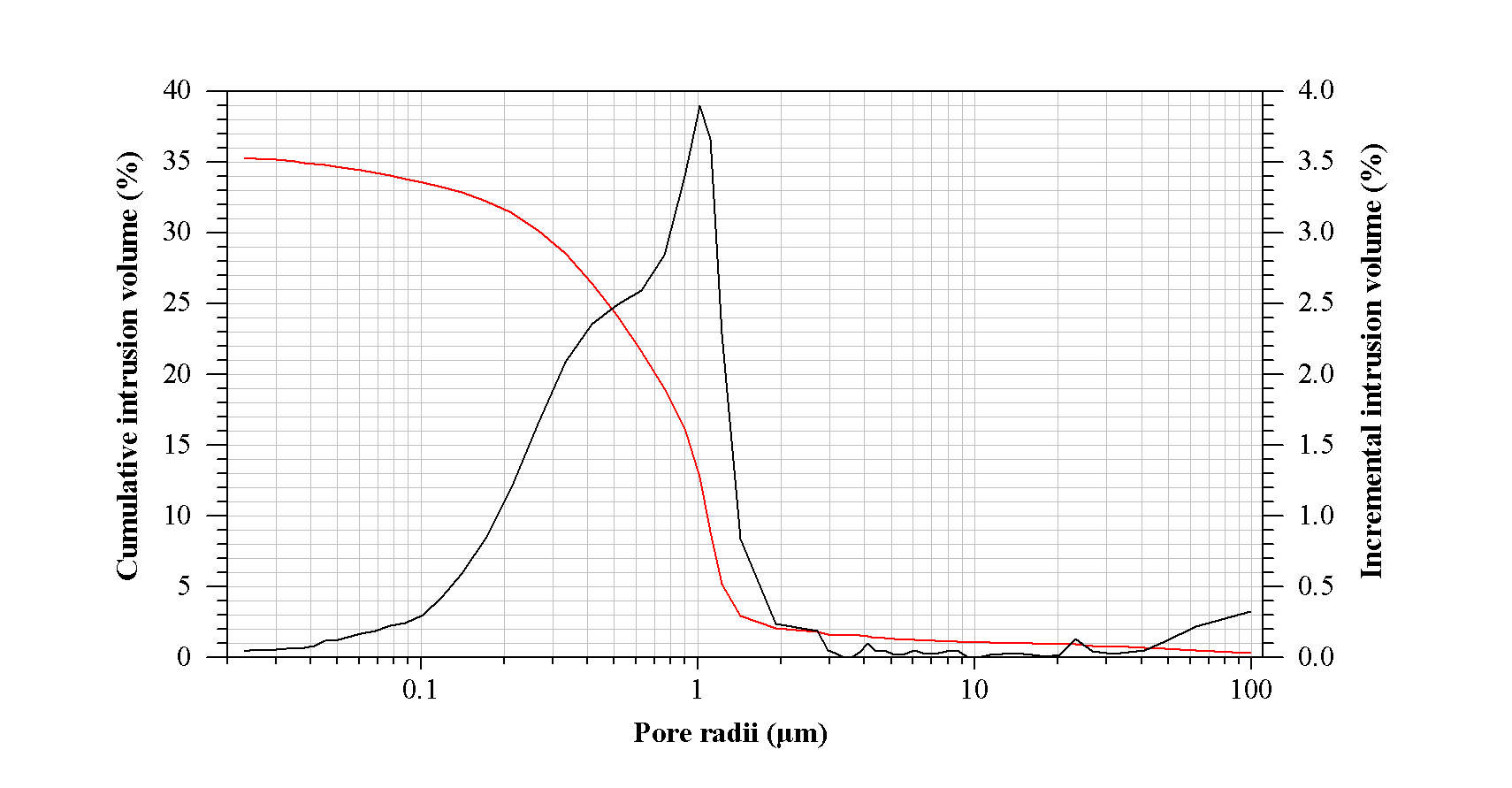


Hg-3:


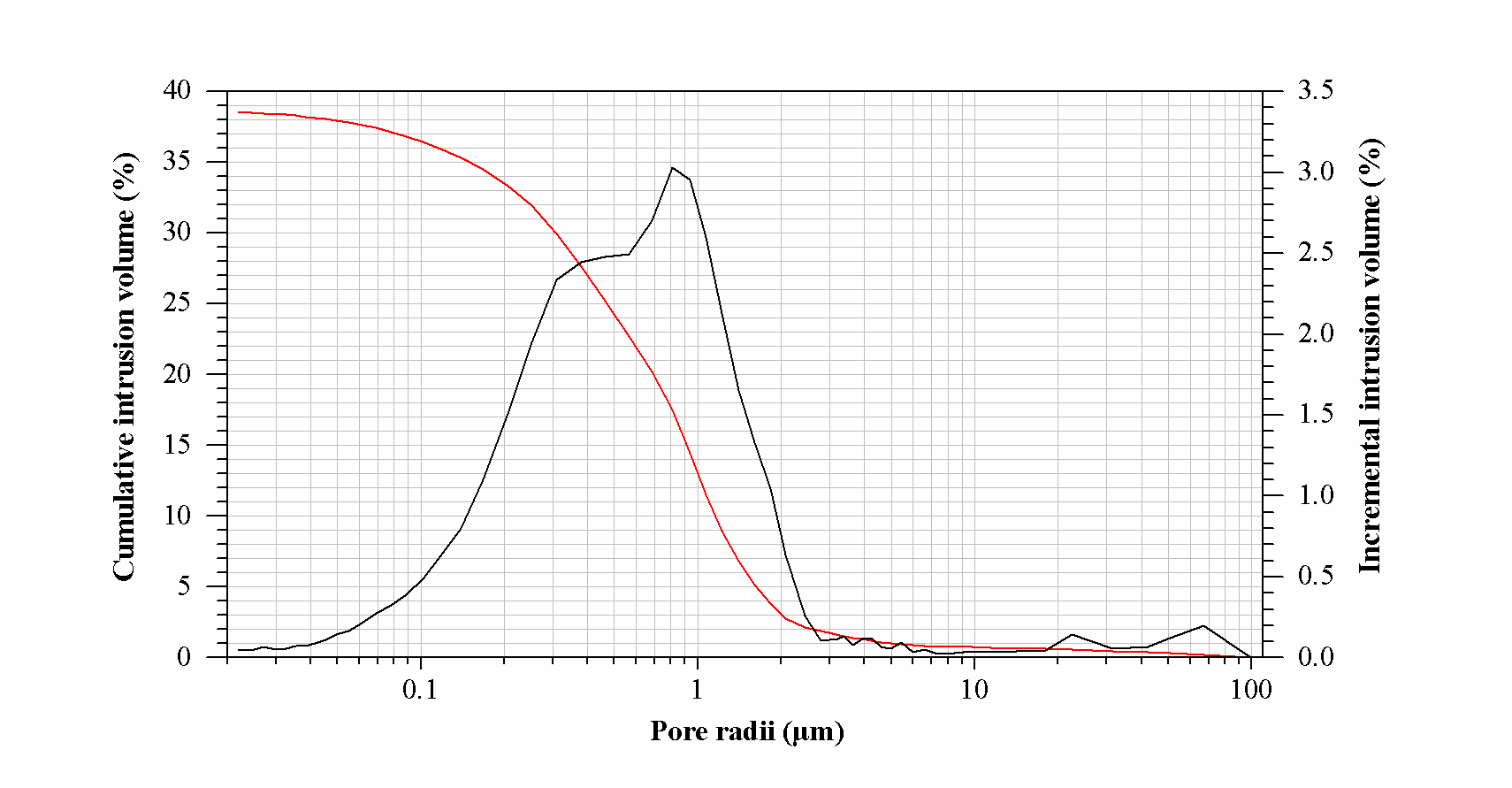


Hg-4:


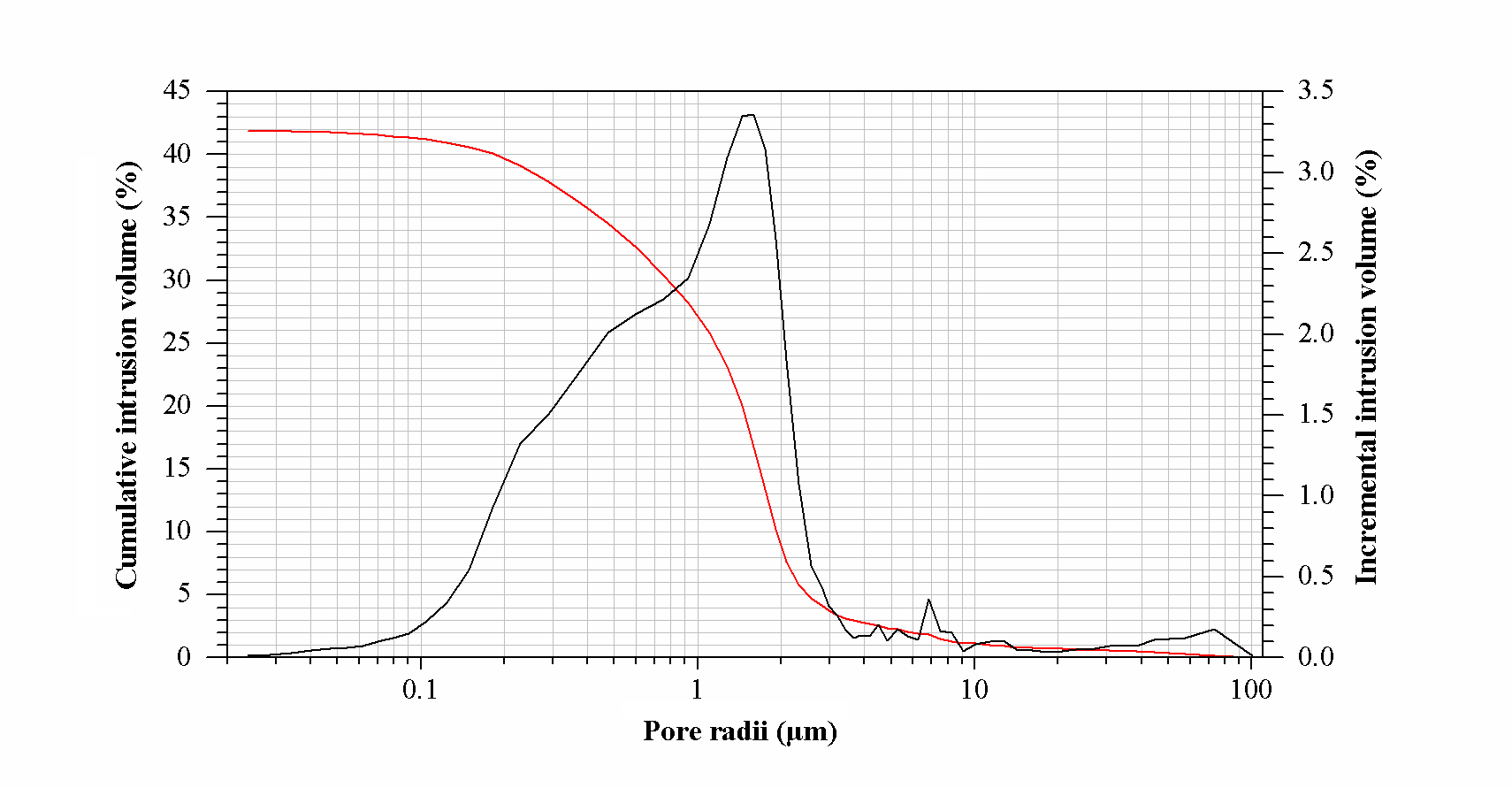


Hg-5:





Mortar:


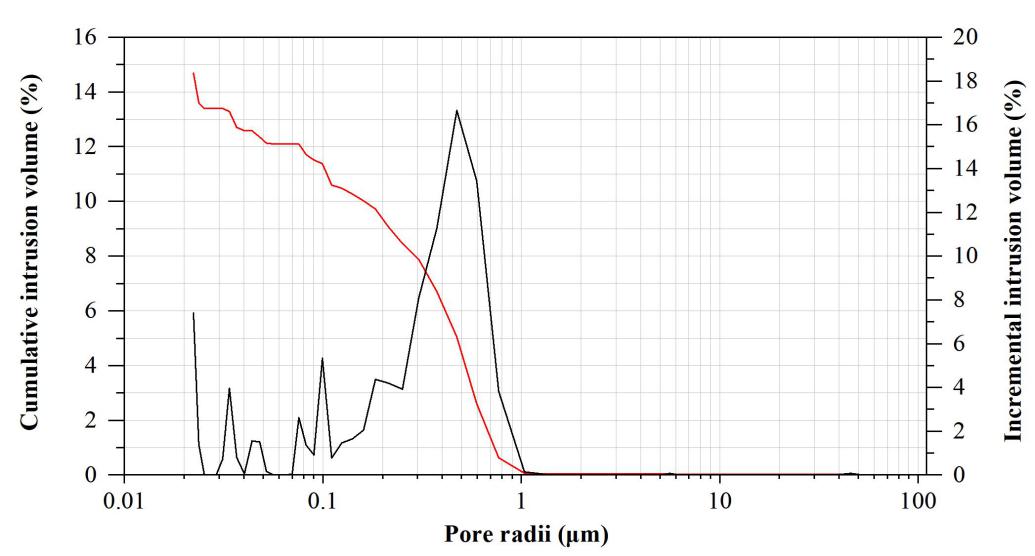


**Fig. S2 PSD curves of six samples derived from compact Ping Yao ancient bricks or mortar.**

**S3 Failure photos and UCS values.**


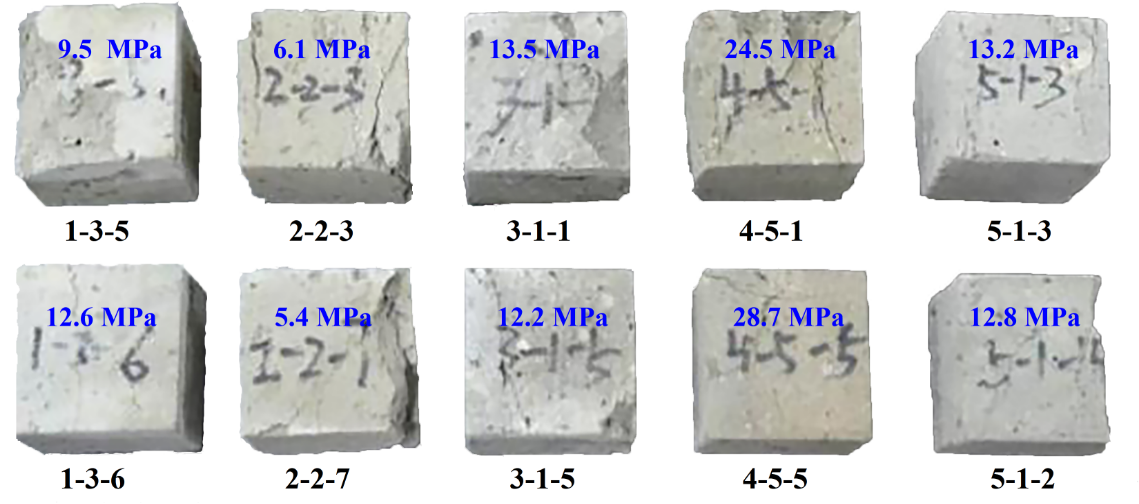


**Fig. S3 Failure photos and values of the UCS (see blue numbers in the picture) of the ten compact samples derived from Ping Yao ancient bricks**

**S4 Plots used for experimental determination of the maximum firing temperatures.**

| 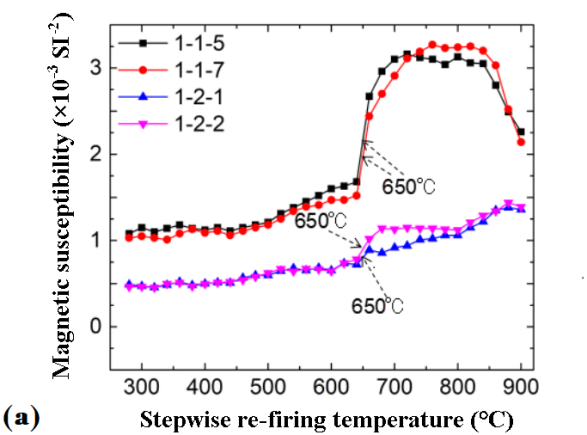 | 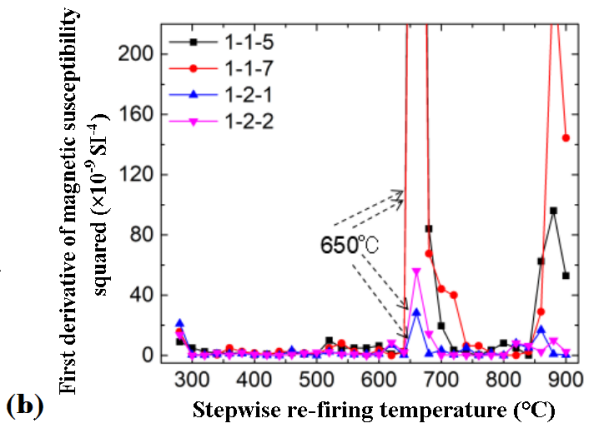 |
| --- | --- |
| **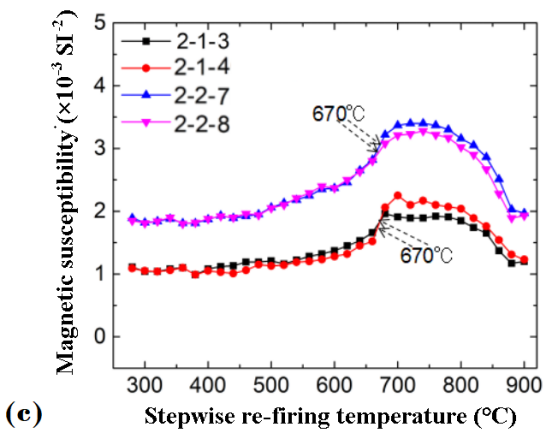** | 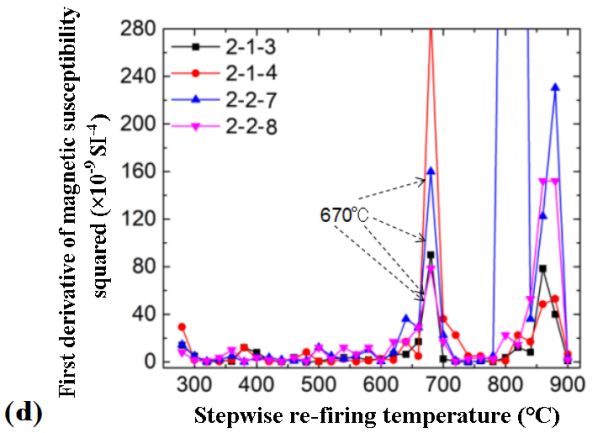 |
| 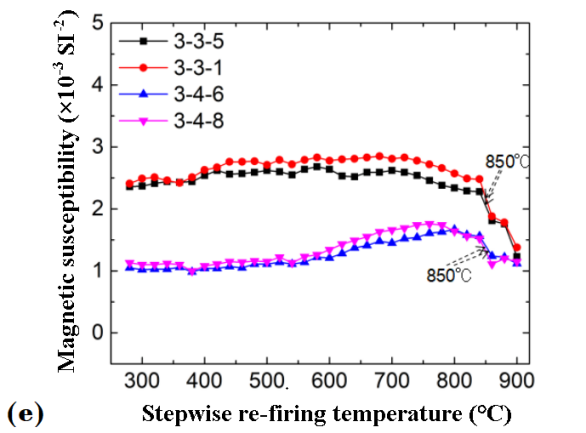 | 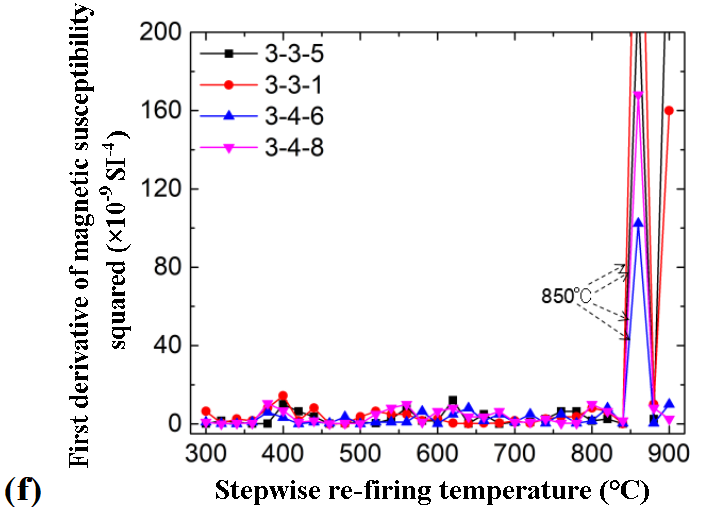 |
| 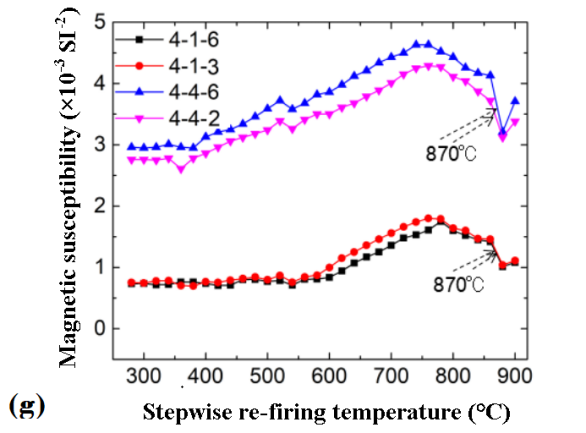 | 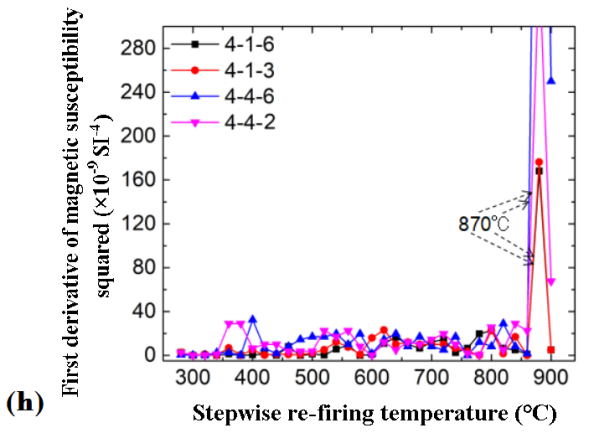 |
| 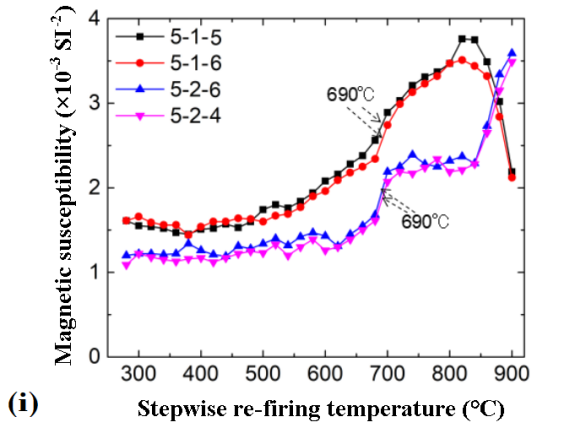 | 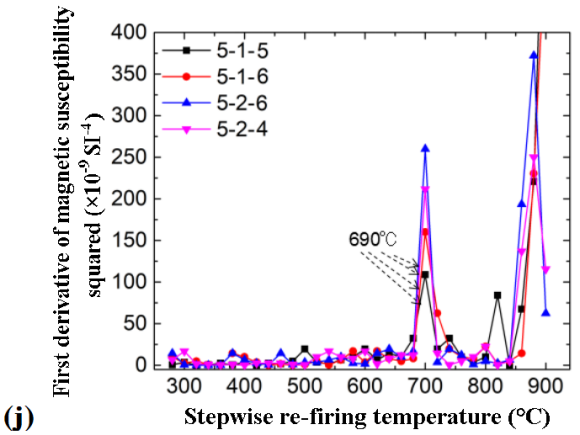 |

**Fig. S4 Plots used for experimental determination of the maximum firing temperatures of twenty samples derived from ten compact Ping Yao ancient bricks.**

**S5 Temperature and rainfall/snowfall during moisture testing period.**


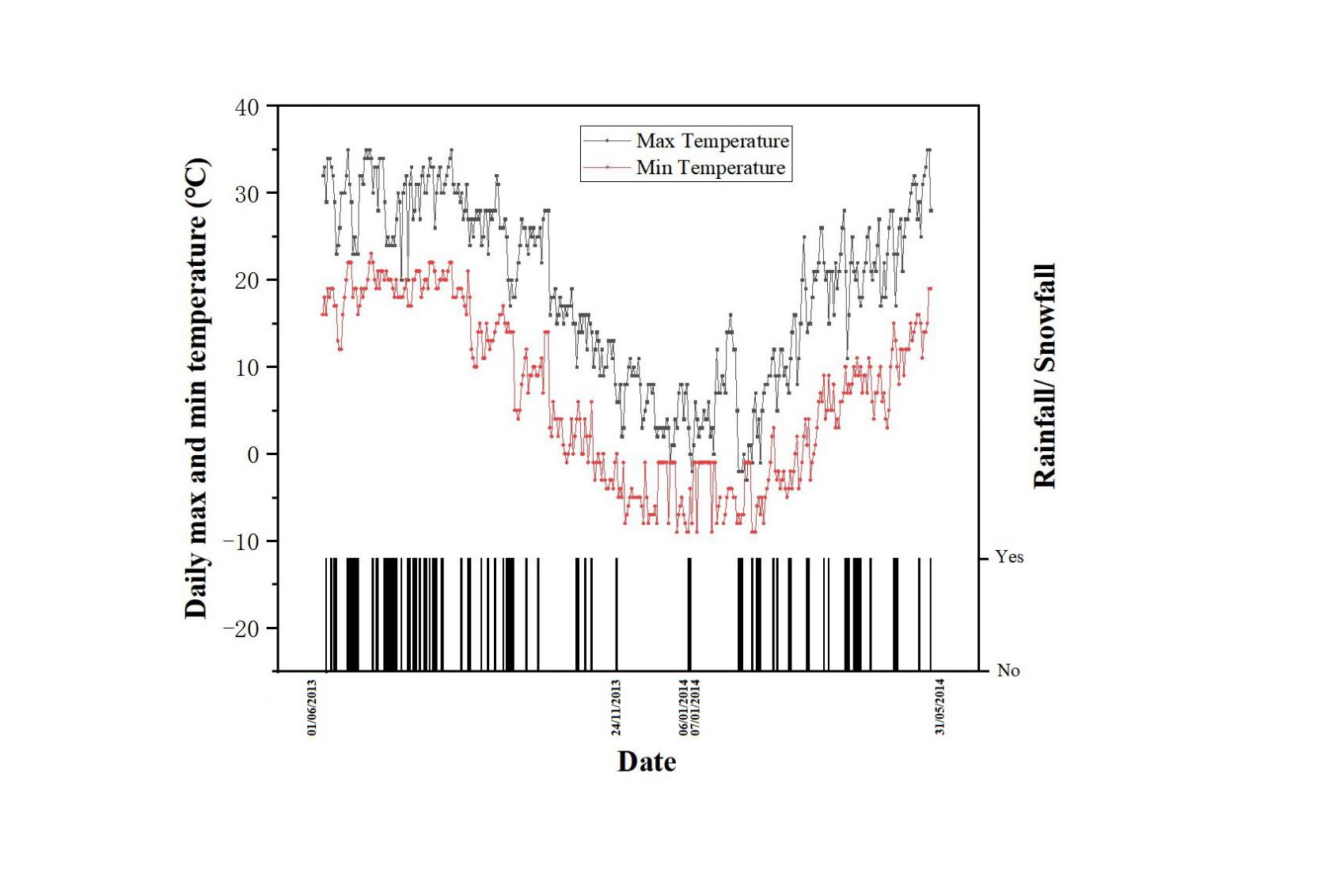


**Fig. S5 Plot of temperature and rainfall/snowfall during moisture testing period.**
